# Biomarker candidates for oocyte and embryo quality assessment in assisted reproduction: A systematic review

**DOI:** 10.5935/1518-0557.20250043

**Published:** 2025

**Authors:** Eloiza Adriane Dal Molin, Virgínia Meneghini Lazzari, Fernanda Souza Peruzzato, Evelise Maria Nazari, Yara Maria Rauh Müller

**Affiliations:** 1 Programa de Pós-graduação em Biologia Celular e do Desenvolvimento, Universidade Federal de Santa Catarina, Florianópolis, SC, Brasil; 2 Laboratório de Reprodução e Desenvolvimento Animal (LRDA), Centro de Ciências Biológicas, Universidade Federal de Santa Catarina, Florianópolis, SC, Brasil; 3 Departamento de Biologia Celular, Embriologia e Genética, Centro de Ciências Biológicas, Universidade Federal de Santa Catarina, Florianópolis, SC, Brasil

**Keywords:** assisted reproductive technology, follicular fluid, granulosa cell.

## Abstract

In assisted reproductive technologies, a major challenge is the relatively low overall success rate, mainly due to the absence of reliable markers for assessing oocyte and embryo quality. This study focuses on identifying new biomarkers in follicular fluid and granulosa cells to better understand the factors essential for oocyte maturation. We followed the 2020 PRISMA guidelines to review recent studies from PubMed, Scopus, and Embase, dated 2014-2024. Our review included 3062 participants from 29 studies, which identified key biomarkers in follicular fluid, such as melatonin and interleukin-10, and in granulosa cells, including mitochondrial DNA and specific transcriptomic signatures. These studies also highlighted the importance of morphological variables and DNA methylation in oocyte quality assessment. Our findings suggest that oxidative stress evaluation in follicular fluid and transcriptomic analysis of granulosa cells are promising approaches for determining oocyte quality in assisted reproduction. This knowledge has the potential to enhance patient care and optimize treatment outcomes in this field.

## INTRODUCTION

Since the birth of the first child conceived through *in vitro* fertilization (IVF) in 1978, the field of reproductive medicine has experienced significant advancements (Severino & Póvoa, 2021). The global demand for assisted reproductive technology (ART) continues to surge (Beilby *et al.*, 2023), as evidenced by the European Society of Human Reproduction and Embryology (ESHRE) reporting the delivery of over 10 million babies through ART (Feuer *et al.*, 2014). Despite these achievements, the success rate of IVF remains relatively low, with live birth rates ranging from 30%-40% (Sang *et al.*, 2021). Within this context, these diminished success rates can be partially attributed to the absence of reliable molecular markers to assess oocyte and embryo quality, along with recurring challenges such as interruption of oocyte maturation, fertilization failure, impaired embryonic formation, and implantation difficulties (Patrizio *et al.*, 2007; Sang *et al.*, 2021; Sciorio *et al.*, 2022).

Failures in human oocyte competence arise from multifaceted factors, including intrinsic oocyte quality (Sciorio *et al.*, 2022). In this context, dynamic interactions in the follicular microenvironment play a pivotal role. Cumulus cells (CC) or granulosa cells (GC), a subset of the follicular cells, intricately orchestrate essential cytoplasmic processes that are crucial for oocyte maturation and quality (Sciorio *et al.*, 2022). These activities are vital for carbohydrate-protein production, organelle positioning, and metabolic pathways necessary for oocyte maturation, competence, and successful fertilization (Patrizio *et al.*, 2007; Sciorio *et al.*, 2022).

While oocyte morphological classification predominantly relies on microscopy techniques, it is important to note that some techniques might introduce a degree of evaluator subjectivity and possess inherent limitations (Ruvolo *et al.*, 2013; Sirait *et al.*, 2021; Lemseffer *et al.*, 2022; Sciorio *et al.*, 2022). Certain methods may not identify critical nuclear and cytoplasmic processes as well as signaling pathways in the cumulus-oocyte complex (COC) regulatory loop (Ruvolo *et al.*, 2013; Sirait *et al.*, 2021). Consequently, there is a need to explore non-invasive methods that can complement morphological analysis and identify quality predictors, thereby enhancing oocyte evaluation and improving ART outcomes (Fischer *et al.*, 2021). In this context, the identification of candidate biomarkers capable of evaluating oocyte quality is welcome. Recent studies have focused on investigating non-invasive preditors associated with follicular fluid (FF) and GC, as they offer valuable insights into the molecular and cellular environment of oocytes (Wyse *et al.*, 2020; 2021). These potential biomarkers hold significant promise in augmenting the success rates of ART procedures by enabling a more comprehensive and integrative assessment of oocyte quality.

In this study, we conducted a systematic review of the literature published from 2014 to 2024, The primary goal of this review is to evaluate oocyte quality by identifying potential biomarkers in the literature. This effort seeks to enhance the precision of oocyte assessment, which may lead to improved oocyte selection and better ART outcomes, including higher fertilization rates, increased blastocyst implantation success, and improved overall gestational outcomes. By integrating recent scientific advancements, this study aims to provide relevant findings that could impact future research and clinical practices in reproductive technologies.

## METHODS

In this study, we followed the recommended review framework outlined in the 2020 Preferred Reporting Items for Systematic Reviews and Meta-Analyses (PRISMA) guidelines for the process of literature search, study selection, and data extraction (Page *et al*., 2021).

### Literature Search

To identify articles investigating biomarkers of oocyte quality in ART cases, we conducted a search in major databases related to reproductive medicine, including PubMed, Scopus, and Embase. The search was conducted in March and April 2024, focusing on articles written in English and published between 2014-2024. This ten-year period was chosen to capture the most recent advancements in methodologies and technologies related to biomarker detection and oocyte quality assessment. To optimize our search strategy, we focused on more targeted MeSH terms that are directly related to known predictors and aspects of oocyte quality. These included: (‘oocyte’ AND ‘biomarker’ AND ‘quality’), (‘oocyte biomarker’ AND ‘oocyte quality’ OR ‘oocyte competence’ OR ‘oocyte maturity’) AND (‘polar body’ OR ‘oocyte morphology’ OR ‘meiotic spindle’ OR ‘follicular fluid’ OR ‘granulosa cells’ OR ‘cumulus cells’ OR ‘mitochondria’) as part of our query.

### Literature Selection

Following the search strategy, we excluded ineligible articles and duplicates using Mendeley Reference Manager. Articles not available in full-text or not in English were also omitted. In addition, we filtered out studies on non-human species or those not aligned with our research scope. Additionally, studies involving specific clinical diagnoses such as severe endometriosis and polycystic ovary syndrome (PCOS) were excluded to align with our research objectives. Notably, our study did not specifically select for any particular age group of patients, Body Mass Index (BMI), or specific causes of infertility, including male factors, as inclusion or exclusion criteria. This decision was influenced by the diversity of the studies analyzed. Furthermore, we deliberately opted to include various study designs to encompass a broader spectrum of evidence. Our broader approach enabled us to encompass a wider array of studies, allowing for a more comprehensive evaluation of oocyte quality predictors across various study designs and patient profiles.

After evaluating titles and abstracts, we selected articles that met inclusion criteria for a full-text review. To ensure an assessment of the quality and risk of bias, we utilized the widely accepted Newcastle-Ottawa Scale (NOS), designed for evaluating the methodological quality of non-randomized studies. In the 9-scoring system, we consider studies with an overall score greater than 7 as high quality, followed by scores between 5 and 7 as fair quality and below 5 as low quality. Independently, two reviewers (E.A.D.M., V.M.L.) assessed the quality of the studies based on the NOS criteria. Articles of low quality were excluded from the review, while articles of fair or high quality were analyzed. Discrepancies or disagreements between reviewers were resolved by discussion and consensus.

### Data Extraction

For the studies included in the analysis, data points were collected that included study design, publication date, sample size (number of subjects and/or samples), candidate predictors of oocyte and/or embryo quality, main results, *p*-value, and additional outcomes, if available.

After applying the NOS, an evaluation of the remaining studies was carried out. To enhance the understanding of the methods utilized for oocyte quality evaluation and their implications, we categorized the studies based on the biological samples examined. These categories encompassed parameters of FF, GC, and additional predictors, such as extracellular vesicle-derived microRNAs (EV-miRNAs), morphological assessment, and DNA methylation.

Although some studies have mentioned CC (Scarica *et al.*, 2019; Shen *et al.*, 2020; Wyse *et al.*, 2020; Daei-Farshbaf *et al.*, 2021) or granulosa mural cells (Karabulut *et al.*, 2020), we chose to standardize GC to improve understanding, given that both are follicular cells.

## RESULTS

### Literature Search and Characteristics

**Figure 1:**
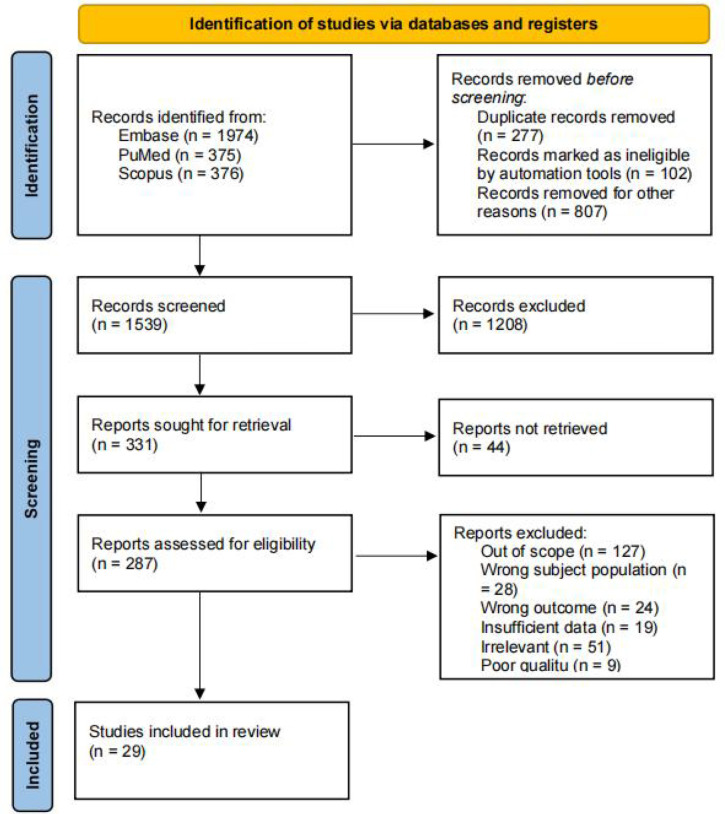
PRISMA 2020 flow diagram for a systematic review investigating biomarkers of oocyte quality.

The databases search resulted in 2725 results. After removing 277 duplicates and identifying articles as ineligible or unavailable for full-text reading using reference management software Mendeley, an additional 807 records were excluded at the title screening phase. These records were eliminated due to various reasons not encompassed by the previous categories, such as encountering animal studies or articles including conditions not pertinent to our inclusion criteria (severe endometriosis and PCOS), as discerned from their titles. We screened 1539 articles based on their titles/abstracts. We excluded 1208 studies due to irrelevance, misaligned objectives, unavailability in full text, or wrong parameters of the subjects. Further evaluation of 287 full-text screens for eligibility resulted in the exclusion of 258 studies, leaving a set of 29 studies (Figure 1).

According to the NOS, 27 out of the 29 articles included in the analysis demonstrated a classification of good quality. However, two articles (Ekapatria *et al.*, 2022; Machtinger *et al.*, 2023) were classified as fair quality due to an imbalance in the sample sizes between the analyzed groups and the lack of vitamin D data, respectively. The final set of studies encompassed various study designs, which are detailed in Tables 1 to 4.

**Table 1:** Biomarkers analyzed in follicular fluid: a summary review results for oocyte evaluation in assisted reproductive technologies.

Study	Study design	Oocyte quality predictor	Samples size	Main outcomes	*p*-value
Carpintero *et al.* (2014)	Prospective cohort	Estradiol, progesterone, testosterone, DHEAS	FF from ICSI patients (n=31)	In FF with MII oocytes, testosterone positively correlated with progesterone (r=0.794 for normal fertilization; r=0.829 for failed fertilization)	<0.0001 both
Santonocito *et al.* (2014)	Crosssectional	miRNAs	FF from ICSI patients (n=15)	37 upregulated in FF compared to plasma, with 32 in microvesicles expressing CD63 and CD81, impacting key follicle growth and oocyte maturation pathways	NA
Bastu *et al.* (2015)	Retrospective cohort	Cathepsin B	FF from IVF patients (n=79)	Cathepsin B levels, total oocytes, MII oocytes, and MII rate were higher in pregnant compared to nonpregnant patients	<0.05 all
Meijide *et al.* (2017)	Prospective case-control	PONs	FF from patients (n=155)	PONs activities were higher in larger follicles (diameter ≥18 mm) than in smaller ones (diameter <12mm);	<0.001
				In donors, PON1 arylesterase and paraoxonase activities showed positive correlations (R^2^=0.078 and R =0.073, resp.)	<0.05 both
Fu *et al.* (2018)	Case-control	miRNAs	FF from ICSI patients (n=91)	MIR-663B was negatively expressed in FF of oocytes that yielded viable blastocysts compared to those that did not	0.019
Bouet *et al.* (2020)	Cross-sectional	Cytokine Profile	FF from IVF/ICSI patients (n=57)	PDGF-BB and RANTES was lower in DOR than NOR;	0.05, 0.023,
				MCP-1 and IL-1RA was elevated in DOR;	0.030, 0.041
				Fewer oocytes in DOR than NOR	0.003
Karabulut *et al.* (2020)	Prospective cohort	TAS; TOS	FF from ICSI patients (n=166)	Mature oocyte rates were decreased in the HOS group	0.002
Latif Khan *et al.* (2020)	Prospective cohort	cfDNA; melatonin	FF (n=895) from IVF patients (n=325)	Intra-follicular cfDNA negatively correlated with elevated melatonin levels in FF samples of mature oocytes	<0.001
Uppangala *et al.* (2020)	Prospective cohort	E2; MDA	FF from ART patients (n=30)	Patients with poor ovarian response and serum E2 <2000pg/mL had elevated MDA levels, with an inverse relationship observed between serum E2 and follicular MDA levels	<0.01 both
Yi *et al.* (2020)	Prospective cohort	Prdx4	FF from IVF/ICSI patients (n=138)	Pdx4 was positively correlated with the oocyte fertilization rate and showed a hight accuracy for clinical pregnancy prediction (AUC= 0.754)	0.011,<0.01, resp.
Wyse *et al.* (2021)	Retrospective	Adiponectin; chemerin; Creactive protein; IL-6; IL-10; IL-18; insulin; leptin; prolactin; resistin; TNF-α; BMP- 15	FF from IVF patients (n=44)	TNF-α and leptin were positive predictors of oocyte maturation (OR=10.2 and 1.2, resp., AUC=0.8);	0.01, 0.04, resp.
				IL-10 was predictive of oocyte maturation after controlling for BMI (OR 1.3x1012; AUC=0.98)	0.02
Ekapatria *et al.* (2022)	Cros-ssectional	Vitamin D	FF (n=77) from IVF patients (n=77)	Vitamin D level in FF was positively correlated with oocyte quality (r=0.32)	0.01
Molka *et al.* (2022)	Prospective and longitudinal	IGF1; GH; IL-6; IL-7	FF from IVF/ICSI patients (n=140)	GH, IGF1, and IL-6 values were higher in group of patients aged ≥ 35 or with development failure or RIF antecedent;	0.001, 0.001, <0.05, resp.
				ROC curve between IL-6 and implantation rate of ongoing pregnancy found an IL-6 cutoff >9.85pg/ml (AUC+0.6)	0.04
Yu *et al.* 2022)	Prospective cohort	LH; FSH; cortisone	FF (n=136) from IVF/ICSI patients (n=138)	FF levels of 17-OHP, testosterone and cortisone level were lower in oocytes of top-quality blastocysts on D5 than the unfertilized group FF DHT concentrations were lower in fertilized follicles after ICSI than in follicles with unfertilized oocytes	<0.05

ART: assisted reproductive technology; BMP-15: bone morphogenetic protein 15; cfDNA: cell-free 913 DNA; DOR: diminished ovarian reserve; E2: estradiol; FF: follicular fluid; FSH: follicle-stimulating hormone; GH: growth hormone; HOS: high oxidative stress; ICSI: intracytoplasmic sperm injection; IGF1: insulin-like growth factor 1; IL: interleukin; IVF: *in vitro* fertilization; LH: luteinize ng hormone; MDA: malondialdehyde; MII: metaphase II oocyte; MCP-1: CCL2; miRNAs: microRNAs; NA: not applicable; NOR: normal ovarian reserve; PONs: paraoxonases; Prdx4: peroxiredoxin 4; RANTES: CCL5; 918 TAS: Total Antioxidant Status; TOS: Total Oxidant Status; TNF-α: tumor necrosis factor-alpha; DHEAS: dehydroepiandrosterone sulfate; DHT: dihydrotestosterone; 17-OHP: 17-hydroxy pregnenolone; D5: day 5; IL-1RA: IL-1 receptor antagonist; PDGF-BB: platelet-derived growth factor-BB.

**Table 2:** Biomarkers analyzed in granulosa cells: a summary review results for oocyte evaluation in assisted reproductive technologies.

Study	Study design	Oocyte quality predictor	Samples size	Main outcomes	*p*-value
Bouckenheimer *et al.* (2018)	Retrospective with prospective validation	GC and MII oocytes lncRNAs	MII oocytes (n=10), GC (n=10) from human samples (n=135)	lncRNAs BCAR4, TUNAR, CASC8, C3orf56, LINC01118, and OOEP-AS1 were unique to MII oocytes, whereas ANXA2P2, IL6STP1, and MALAT1 were exclusive to GC	<0.05
Scarica *et al.* (2019)	Prospective exploratory	PTGS2; CAMK1D; HAS2; STC1; EFNB2	GC (n=75) from ART patients (n=18)	CAMK1D, PTGS2, and HAS2 levels were lower in GC isolated from oocytes resulting in developmental arrest compared to reaching the blastocyst stage	0.01, 0.05, 0.03 resp.
Karabulut *et al.* (2020)	Prospective cohort	Hsp70; Tgf-β; Notch 1	GC from ICSI patients (n=166)	Hsp70 and Notch1 were lower expressed in the HOS group Chromatin integrity was decreased in the HOS group	0.0026, 0.0047 resp 0.0023.
Shen *et al.* (2020)	Prospective observational	PFKP; PKM2; LDHA; GFPT; HAS2; VCAN; TNFAIP6; PTGS2; PTX3; SDC4	GC (n=354) from women (n=48)	GC PFKP expression from mature oocytes was higher than those from immature oocytes	0.014
Uppangala *et al.* (2020)	Prospective	P53; HSP70; OGG1	GC from ART patients (n=30)	P53, HSP70, and OGG1 expression in GC was elevated in poor responders	<0.05-0.01
Wyse *et al.* (2020)	Prospective cohort	Transcriptomic signatures	GC from IVF/ICSI patients (n=18)	Transcriptomic signatures representing GC of different maturity had 1818 DEGs; GC encapsulating mature oocytes had 2407 fewer transcripts detected than in GC of immature oocytes	NA
Cecchino *et al.* (2021)	Prospective cohort	Bioenergetic properties	GC from IVF/ICSI Patients (n=127)	GC from > 35 women had lower mitochondrial respiration, aerobic glycolysis, and cellular energy charge	0.046, 0.009, 0.001, resp.
Daei-Farshbaf *et al.* (2021)	Retrospective with prospective validation	PPP3	GC from ICSI patients (n=20)	PPP3CB expression level was elevated in GC of fertilized MII oocytes compared to immature and unfertilized MII oocytes	≤0.05
Martins *et al.* (2022)	Laboratory based experimental	Proand antiinflammatory mediators	GC from ART patients undergoing ovarian stimulation (n=32)	Pro-resolving mediators produced by M1 and M2 macrophages in GC can partially reverse ovarian inflammation by increasing the expression of 12-LOX and COX-2, resp.	<0.05

12-LOX: 12-lipoxygenase; ART: assisted reproductive technology; CAMK1D: calcium/calmodulin-dependent protein kinase 1D; DEGs: differentially expressed genes; EFNB2: ephrin-B2; GC: granulosa cells; GFPT: glutamine fructose-6- phosphate transaminase; GH: growth hormone; HAS2: hyaluronan synthase 2; HOS: high oxidative stress; Hsp70: Heat shock protein 70; ICSI: intracytoplasmic sperm injection; IL6STP1: interleukin 6 signal transducer pseudogene 1; IVF: *in vitro* fertilization; LDHA: lactate dehydrogenase A; lncRNAs: long non-coding RNAs; MALAT1: metastasis associated lung adenocarcinoma transcript 1; MII: metaphase II; NA: not applicable; Notch1: notch homolog 1; OGG1: 8-oxoguanine DNA glycosylase 1; P53: tumor protein 53; PFKP: phosphofructokinase platelet; PKM2: pyruvate kinase M2; PPP3: protein phosphatase 3; PTGS2: prostaglandin-Endoperoxide Synthase 2; PTX3: pentraxin 3; SDC4: syndecan 4; STC1: stanniocalcin 1; TAS: Total Antioxidant Status; Tgf-β: Transforming growth factor-beta; TNFAIP6: tumor necrosis factor alpha-induced protein 6; VCAN: versican.

### Patient Profile

Our systematic review encompassed 3,062 individuals from 29 studies, highlighting substantial heterogeneity in participant inclusion and exclusion criteria, particularly regarding age, infertility causes, and BMI.

#### Age Criteria

Age-based inclusion criteria varied widely across studies. Some studies exclusively focused on younger participants, such as Ekapatria *et al.* (2022), Santonocito *et al.* (2014), Meijide *et al.* (2017), McCulloh *et al.* (2020), Uppangala *et al.* (2020), and Yi *et al.* (2020), which limited inclusion to participants under 35 years. Others imposed stricter or broader limits: Karabulut *et al.* (2020) included only those aged 25-37, Machtinger *et al.* (2023) set the upper age limit at 36, and Yu *et al.* (2022) excluded individuals above 38 years. Broader age ranges were observed in studies like Shen *et al.* (2020) (22-42 years), Molka *et al.* (2022) (19-42 years), and Martinez *et al.*, (2019) (19-38 years). Conversely, some studies focused on older populations, such as Scarica *et al.* (2019), Cecchino *et al.* (2021), and Tepla *et al.* (2022), which primarily included women over 35 years. Additionally, some studies did not specify age criteria (Bouckenheimer *et al.*, 2018; Latif Khan *et al.*, 2020; Daei-Farshbaf *et al.*, 2021; Yuan *et al.*, 2021; Zhang *et al.*, 2021a), while others only reported mean participant ages, such as 33-35 years (Wyse *et al.*, 2020; 2021) and 32 years (Martins *et al.*, 2022).

#### Causes of Infertility

The reviewed studies also varied in how male infertility was considered. Some studies applied strict exclusion criteria for male infertility, explicitly excluding participants with severe male factor infertility (Martinez *et al.*, 2019; Scarica *et al.*, 2019; Zhang *et al.*, 2021a; Molka *et al.*, 2022; Yu *et al.*, 2022), while Fu *et al.* (2018) specifically excluded azoospermia, severe oligozoospermia, and teratozoospermia. In contrast, other studies imposed no restrictions on male factor infertility, including Carpintero *et al.* (2014), Bastu *et al.* (2015), Meijide *et al.* (2017), and Wirleitner *et al.* (2018).

Some studies explicitly focused on male infertility factors (Santonocito *et al.*, 2014; Latif Khan *et al.*, 2020; Shen *et al.*, 2020; Yi *et al.*, 2020; Cecchino *et al.*, 2021; Martins *et al.*, 2022; Machtinger *et al.*, 2023). Shen *et al.* (2020) identified asthenozoospermia and oligozoospermia as primary infertility causes, while Machtinger *et al.* (2023) reported that 41.8% of cases were due to male factors. Similarly, Latif Khan *et al.* (2020) found that male factor infertility accounted for 57.5% of cases, and Meijide *et al.* (2017) observed that some type of male infertility- often in combination with other causes-was present in 58% of cases. Yi *et al.* (2020) and Latif Khan *et al.* (2020) further noted that male age and BMI did not significantly differ between pregnant and non-pregnant groups. On the other hand, some studies provided limited or no information on male infertility, such as Wyse *et al.* (2020; 2021) and Tepla *et al.* (2022), while Bouckenheimer *et al.* (2018) and Bouet *et al.* (2020) did not specify infertility causes.

#### BMI

Several studies applied BMI-based exclusions, such as He *et al.* (2023) and Yi *et al.* (2020), which excluded participants with BMI ≥24 kg/m^2^, while Sheng *et al.* (2017) and Ekapatria *et al.* (2022) only included individuals with BMI <25kg/m^2^. Similarly, (Machtinger *et al.*, 2023) restricted inclusion to women with a normal or high-normal BMI (BMI < 28 kg/m^2^), and Yu *et al.* (2022) analyzed a cohort with BMI ranging from 17.2 to 27.7kg/m^2^. Other studies, such as Meijide *et al.* (2017), Daei-Farshbaf *et al.* (2021), Karabulut *et al.* (2020), and Uppangala *et al.* (2020), focused on participants within specific BMI ranges, excluding those above 26kg/m^2^.

Beyond eligibility criteria, BMI was also linked to molecular variations in CC and FF composition. Shen *et al.* (2020) reported that patient age and BMI influenced gene expression patterns in CC, while Wyse *et al.* (2021) identified a consistent correlation between TNF-α and BMI, with a disruption of leptin regulation at BMI ≥28kg/m^2^. Additionally, Wyse *et al.* (2021) found that, when stratified by BMI, the anti-inflammatory cytokine IL-10 and the pro inflammatory cytokine IL-18 were predictive of oocyte maturation in women with normal BMI, with IL-10 remaining significant even after controlling for BMI.

### Biomarkers Found in Follicular Fluid

Among the 29 articles included in the analysis, 14 evaluated various aspects of the FF environment, such as the levels of Vitamin D, growth hormone (GH) or melatonin supplementation, cell-free DNA (cfDNA) fragments, as well as inflammatory parameters, biomarkers of oxidative stress, steroid levels, and regulation of immune responses within the FF (Table 1).

### Biomarkers Within Granulosa Cells

We identified 9 articles that focused on several parameters associated with GC, including the evaluation of diverse aspects such as bioenergetic properties, apoptotic indices, enzymatic regulation, mitochondrial DNA (mtDNA) copy numbers, proand anti-inflammatory mediators, and the transcriptomic profile of GC (Table 2).

**Table 3:** Biomarkers encompassing miRNAs, morphology parameters, DNA methylation, and patient characteristics: a summary of review results for oocyte evaluation in assisted reproductive technologies.

Study	Study design	Oocyte quality predictor	Samples size	Main outcomes	p-value
Wirleitner *et al.* (2018)	Prospective cohort	Follicle size	Follicles from IVF patients (n=118)	Oocyte recovery was lower in small follicles (63.8%) *versus* medium (76.6%) and large (81.3%); Estradiol and progesterone levels were higher in large compared to medium and small follicles	0.001, 0.01, 0.05, resp.
Martinez *et al.* (2019)	Cross-sectional	Phenols and phthalates; EVmiRNAs	FF EV-miRNA from IVF patients (n=130)	Select urinary phenol and phthalate biomarkers are associated with altered expression of eight FF EV-miRNAs	<0.05
McCulloh *et al.* (2020)	Prospective cohort	Follicle size	Follicles from oocyte donors (n=22)	The incidence of MII oocytes was significantly associated with larger follicle size (mm) (ROC’s AUC=0.87)	<0.0001
Yuan *et al.* (2021)	Prospective experimental	LINE, LTRs, and methylation alterations in PBI promoter	PBIs and sibling MII oocytes (n=74) from donors (n=6)	PBI methylome aligns closely with sibling MII oocyte (r=0.92); elevated LINE & LTR methylation noted in PBIs not progressing to blastocyst	<0.05 both
Zhang *et al.* (2021a)	Prospective observational	EV-miRNAs; BDNF	FF EV-miRNA from ART patients (n=56)	BDNF mRNA and protein levels were higher in FF from mature follicles;	<0.01, <0.05, resp.
				miR-103a-3p and miR-10a-5p expressions had negative correlations with BDNF mRNA levels in FF	<0.01 both
Bartolacci *et al.* (2022)	Systematic review	Oocyte dismorphisms	Systematic Review (study size=76)	Negative outcomes were associated with COC Morphology, SER, vacuoles, and granulometry with an OLS 8.33,7.78, 7.14, and 6.25, resp.	NA
Tepla *et al.* (2022)	Prospective observacional cohort	MS visibility and relative position to the PB on oocyte maturity	IVF patients (n=679)	Patients >35 years: 32% PR 58/182) with and 24% PR (47/195) without MS visualization	<0.1 both
Machtinger *et al.* (2023)	Prospective cohort	r-hCG, GnRH-a, EV-miRNA	FF EV-miRNA from IVF patients (n=91)	r-hCG stimulation protocol group had fewer oocytes retrieved and fewer mature oocytes than the GnRH-a group;	0.02 both
				41 EV-miRNAs expression levels significantly differed between the two protocols	<0.05

ART: assisted reproductive technology; BDNF: brain-derived neurotrophic factor; COC: cumulus-oocyte complex; EV miRNA: extracellular vesicle microRNA; FF: follicular fluid; GnRH-a: gonadotropin-releasing hormone agonist; IVF: *in vitro* fertilization; LINE: hypermethylated long interspersed nuclear element; LTRs: long terminal repeats; MII: metaphase II; microRNA: micro RNA; MS: meiotic spindle; NA: not applicable; OLS: oocyte literature score; PB: polar body; PR: pregnancy rate; r-hCG: recombinant human chorionic gonadotropin; ROC: receiver operating characteristic; SER: smooth endoplasmic reticulum aggregates.

**Table 4:** Oocyte quality biomarkers in embryo assessment: a summary of review results for oocyte evaluation in assisted reproductive technologies.

Study	Study design	Time of assessment	Developmental quality predictor	Developmental stage grading	Main outcomes	p-value
Carpintero *et al.* (2014)	Prospective cohort	D3	Estradiol; progesterone; testosterone	Ardoy *et al*. (2008)	Embryo qualities A and C, and B and C, differed in the estradiol/progesterone ratio;	0.003, 0.0009
					Embryo qualities B and C differed in the estradiol/testosterone ratio	0.001
Wirleitner *et al.* (2018)	Prospective cohort	D5	Follicle size	Gardner *et al*. (2000)	Blastocyst rates were lower in small than in medium follicles, both per aspirated follicle (15.3% vs 27.9%) and Per COC (24.0% vs 36.4%);	<0.05 Both
					The rate of top-quality blastocysts was 12.1% in small, 19.4% in medium and 14.3% in large follicles	NS
Fu *et al.* (2018)	Case-control	D5	MIR-663B	Top-scoring: blastocysts with good ICM and TE assessed as A or B (≥3BB); poor-scoring: blastocysts with few and large cells for ICM or TE assessed as C (<3BB)	Expression levels of FF MIR-663B for top-scoring embryos were lower than that of poor scoring	0.003
Latif Khan *et al.* (2020)	Prospective cohort	D3	cfDNA; melatonin	Morphological criteria: i) cleavage rate and the number of blastomeres, ii) cytoplasmic appearance, iii) extent of fragmentation, iv) regularity in the symmetry of blastomeres	cfDNA was lower in FF related to oocytes that produced topquality embryos compared to low quality embryos	<0.001,
					In non-pregnant women, oocytes from FF with lower melatonin levels resulted in embryos with higher fragmentation rates	< 0.001
McCulloh *et al.* (2020)	Prospective cohort	D5; D6	Follicle size	Gardner *et al*. (2000)	Quality blastocysts were found to have an association with larger follicles	0.01
Scarica *et al.* (2019)	Prospective exploratory	Before TE biopsy	PTGS2; CAMK1D; HAS2	Gardner & Schoolcraft (1999)	CAMK1D, PTGS2, and HAS2 were more highly expressed in GC of oocytes that resulted in embryos reaching the blastocyst stage than in those that experienced developmental arrest	0.01, 0.05, 0.03, resp.
Shen *et al.* (2020)	Prospective observational	D3	VCAN	Gardner & Sakkas (2003)	VCAN was positively correlated to embryo grade	0.024
Uppangala *et al.* (2020)	Prospective cohort	D3	E2; MDA	Morphological criteria: grade I corresponded to stage-specific cell number, uniform blastomere size, without multinucleation, and no or <10%fragmentation	Patients with normal ovarian response and serum E2 between 2000-3000 pg/mL had 77.03 % grade I embryos;	<0.01 both
					Patients with lower and higher levels had 40.74% and 51.88% compared to normal, resp.	< 0.01
Yi *et al.* (2020)	Prospective cohort	D3	Prdx4; SOD	Good quality embryos contained at least 7 cells and with ≤ 10% of fragmentation	Prdx4 and SOD levels were positively correlated with good quality embryo rate	0.013, 0.021
Yuan *et al.* (2021)	Prospective experimental	T2; t4; t8; D5; D6	LINE; LTRs	Not specified	DNA methylation in LINE (including L1, L2) and LTR was higher in PBIs of embryos that could not reach the blastocyst stage	<0.05
Zhang *et al.* (2021a)	Prospective observational	D3; D5	miR-103a-3p; miR-10a-5p	Good quality embryos developed from normal fertilized eggs with no or ≤ 1/3 fragmentation	miR-103a-3p and miR-10a-5p expression levels were increased in the subgroups in which oocytes did not develop into high-quality embryos on D3 or D5	D3: 0.05, 0.01, resp. D5: 0.05 both
Yu *et al.* (2022)	Prospective cohort	D3;D5	LH;FSH; cortisone	High-quality embryos had 8 symmetrical, non-fragmented blastomeres on D3, and showed the development of ICM, the appearance of the TE, and the expansion of the blastocyst on D5	FF LH, FSH, and cortisone were strong predictors of top-quality embryos on D5	0.037, 0.014, 0.04, resp.
Machtinger *et al.* (2023)	Prospective cohort	D3	r-hCG; GnRH-a	Top-quality embryo contained 7-8 equal blastomeres and ≤10% of fragmentation	There was no significant difference in the number of top-quality embryos between the r-hCG and GnRH-a stimulation protocol groups	NS

CAMK1D: calcium/calmodulin-dependent protein kinase ID; cfDNA: cell-free DNA; COC: cumulus-oocyte complex; D2: day 2; D3: day 3; D5: day 5; D6: day 6; E2: estradiol; FF: follicular fluid; FSH: follicle stimulating hormone; GC: granulosa cells; GnRH-a: gonadotropin-releasing hormone GnRH agonist; HAS2: hyaluronan synthase 2; ICM: inner cell mass; LH: luteinizing hormone; LINE: long interspersed nuclear element; L1: LINEI; L2: LINE2; LTR: long terminal repeats; MDA: malondialdehyde; MIR-663b: microRNA-663B; NS: not significant; PBI: polar body I; Prdx4: peroxiredoxin 4; PTGS2: prostaglandin-endoperoxide synthase 2; r-hCG: recombinant human chorionic gonadotropin; SOD: superoxide dismutase; TE: trophectoderm; t2: two-cell stage; t4: four-cell stage; t8: eight-cell stage; VCAN: versican.

### Additional Parameters in Oocyte Quality Assessment: EV-miRNAs, Morphological Features, and DNA Methylation

Our investigation uncovered three studies (Martinez *et al.*, 2019; Zhang *et al.*, 2021a; Machtinger *et al.*, 2023) that utilized advanced in silico analyses to explore EV-miRNA activities and two (McCulloh *et al.*, 2020; Bartolacci *et al.*, 2022) that assessed oocyte morphology parameters’ impact on oocyte development. Additionally, we found one study Tepla *et al.* (2022) that examined the relationship between meiotic spindle (MS) morphology and its positioning relative to the polar body, shedding light on its critical role in oocyte maturation. We also identified one study (Zhang *et al.*, 2021a) that focused on DNA methylation changes in metaphase II (MII) oocytes with diminished potential for embryonic development, unraveling the epigenetic complexities that underlie oocyte competence. Furthermore, Wirleitner *et al.* (2018) focused on the relationship between follicle size and oocyte quality, blastocyst development, and live-birth rate, identifying the optimal follicle size for oocyte retrieval (Table 3).

### Impact of Oocyte Quality Biomarkers on Further Outcomes

Among the 29 articles analyzed, 17 investigated the correlation between their findings and the potential for oocyte fertilization and/or fertilization rates (Carpintero *et al.*, 2014; Bastu *et al.*, 2015; Meijide *et al*., 2017; Fu *et al.*, 2018; Wirleitner *et al.*, 2018; Karabulut *et al.*, 2020; Latif Khan *et al.*, 2020; McCulloh *et al.*, 2020; Shen *et al.*, 2020; Uppangala *et al.*, 2020; Yi *et al.*, 2020; Daei-Farshbaf *et al.*, 2021; Wyse *et al.*, 2021; Yuan *et al.*, 2021; Ekapatria *et al.*, 2022; Molka *et al.*, 2022; Yu *et al.*, 2022).

In the context of fertilization rates and FF biomarkers, Karabulut *et al.* (2020) found a correlation between the oxidative status of FF and fertilization rates (*p*=0.001, r=0.518). Similarly, Yi *et al.* (2020) demonstrated a positive correlation between FF superoxide dismutase (SOD) levels and the fertilization rate (r=0.307; *p*=0.020); Yu *et al.* (2022) indicated that FF dihydrotestosterone (DHT) levels may predict unsuccessful fertilization (sensitivity of 0.82, specificity of 0.65), while Wyse *et al.* (2021) recognized prolactin in FF as the key predictor for fertilization rates (sensitivity of 0.77, specificity of 0.78).

Carpintero *et al.* (2014) reported higher FF progesterone levels during normal fertilization compared to failed fertilization (*p*=0.003). Additionally, Bastu *et al.* (2015) found that FF Cathepsin B levels correlated with fertilization rates (*p*=0.042). Moreover, Meijide *et al.* (2017) observed significant differences in FF PON3 activity between donors and patients in large follicles, with a 20.7% higher level in donors (16.3±1.0 *vs*. 13.5±0.5nmol/min/ml), remaining significant after adjusting for age, BMI, number of retrieved oocytes, and fertilization rate (*p*=0.020).

In terms of other biomarkers and fertilization rates, McCulloh *et al.* (2020) stated that the follicle diameter does not effectively predict fertilization. Yuan *et al.* (2021) noted significant increases in the expression of LINE and LTR post-fertilization in the first polar body (PB1) and MII oocyte pair. Meanwhile, Shen *et al.* (2020) reported a negative correlation between TNFAIP6 expression in GC and fertilization (*p*=0.044). Additionally, 5 other studies (Wirleitner *et al.*, 2018; Latif Khan *et al.*, 2020; Uppangala *et al.*, 2020; Ekapatria *et al.*, 2022; Molka *et al.*, 2022) found no substantial link between their potential biomarkers and fertilization rates in the studied groups. For development, 13 articles examined biomarkers of embryo development parameters (Table 4).

In the domain of pregnancy outcomes related to oocyte biomarkers, 7 articles investigated this association (Carpintero *et al.*, 2014; Bastu *et al.*, 2015; Wirleitner *et al.*, 2018; Latif Khan *et al.*, 2020; Yi *et al.*, 2020; Molka *et al.*, 2022; Tepla *et al.*, 2022). In this context, Tepla *et al.* (2022) observed that in patients over 35 years of age, the visualization of MS influenced the pregnancy rate. Molka *et al.* (2022) reported a significant association between IGF1 levels and the cumulative pregnancy rate (AUC=0.73, *p*=0.001). Furthermore, they identified correlations between Interleukin-6 (IL-6) and IGF1 levels (r=0.26, *p*=0.005), and between IGF1 levels and endometrial thickness (r=0.37; *p*=0.001).

Carpintero *et al.* (2014) found higher estradiol levels in patients who achieved pregnancy (*p*=0.02). Bastu *et al.* (2015) reported that the area under the curve for cathepsin B predicting pregnancy was 0.662 (*p*=0.024, 95% CI 0.528-0.797). Wirleitner *et al.* (2018) observed similar implantation, pregnancy, clinical pregnancy rates, and live birth rates across transfers of blastocysts from small, medium, or large follicles.

Latif Khan *et al.* (2020) explored cfDNA and melatonin concentrations within FF samples during IVF procedures. Their findings highlighted a marked reduction in cfDNA paired with an elevation in melatonin levels in samples from pregnant women, in contrast to those from non-pregnant subjects (*p*<0.001 for both metrics). Concurrently, Yi *et al.* (2020) elucidated disparities in oxidative stress marker levels, namely GSH-Px (Glutathione Peroxidase), SOD (Superoxide Dismutase), and Prdx4 (Peroxiredoxin 4) in FF samples between pregnant and non-pregnant cohorts (*p*≤0.01). Notably, Prdx4 emerged as a prominent predictor of clinical pregnancy, achieving an AUC of 0.754.

## DISCUSSION

The low rate of successful live births in ART poses a significant challenge in the field (Patrizio *et al.*, 2007; Sang *et al.*, 2021; Sciorio *et al.*, 2022). This may be attributed to factors encompassing inadequate oocyte maturation, failures in fertilization, embryonic development, and/or implantation processes (Patrizio *et al.*, 2007; Pirtea *et al.*, 2021; Sang *et al.*, 2021). As the fate of an embryo is determined at an early stage of development, the corresponding oocyte plays a crucial role in determining the potential for human embryo development (Yuan *et al.*, 2021).

Within the processes of oocyte interaction with its surrounding cells, there appears to be a regulatory dependency on COC, modulating essential functions to establish the optimal microenvironment for oocyte development. Consequently, gene expression profiles in GC as well as FF analyses should also reflect oocyte developmental potential. Therefore, evaluating biomarkers that reflect oocyte quality and examining their influences in predicting embryonic quality are crucial for achieving overall positive outcomes in ART (Belli *et al.*, 2021).

### Biomarkers Within Granulosa Cells: Gene Expression Profiles

HSP70, a conserved member of the heat shock protein family, has been recognized for its potent antioxidant, anti-inflammatory, and anti-apoptotic properties (Uppangala *et al.*, 2020). In this context, studies (Karabulut *et al.*, 2020; Uppangala *et al.*, 2020) have explored the association between HSP70 transcripts and oocyte quality. Karabulut *et al.* (2020) reported lower levels of HSP70 expression in groups experiencing high oxidative stress (HOS), while Uppangala *et al.* (2020) observed a higher expression of transcripts in poor responder patients, characterized by an elevated oxidative stress environment. Although both studies found a correlation between oxidative stress and HSP70 expression, the cause-and-effect relationship is still unclear. While Uppangala *et al.* (2020) propose that HSP70 modulates oxidative stress, Karabulut *et al.* (2020) suggest that oxidative stress suppresses HSP70, leading to a reduction in the oxidative tolerance of the cells.

The hyaluronic acid synthase 2 (HAS2) gene, integral to GC physiology, orchestrates the synthesis of hyaluronic acid, fundamental for the structural integrity of the mature cumulus oophorus (Scarica *et al.*, 2019). In this domain, Scarica *et al.* (2019) correlated enhanced HAS2 transcriptional activity in GC with oocytes that achieved blastocyst morphogenesis, intimating its prospective utility as a prognostic biomarker. Conversely, Shen *et al.* (2020) did not corroborate this correlation across parameters including oocyte maturation, fertilization kinetics, embryonic grading, or implantation success.

Prostaglandin endoperoxide synthase 2 (PTGS2), an enzyme essential for prostaglandin biosynthesis and implicated in cumulus expansion Scarica *et al.* (2019), has yielded intriguing findings. While Shen *et al.* (2020) found no association between PTGS2 and outcomes like oocyte maturity, fertilization, embryo grade, or implantation, Scarica *et al.* (2019) linked PTGS2 strongly with blastocyst development. Additionally, Wyse *et al.* (2020) highlighted PTGS2 as a key factor in GC pivotal for oocyte maturation. These contrasting results highlight the importance of further research with larger cohorts and uniform methodologies to better understand the roles of such biomarkers in oocyte quality.

### Biomarkers Identified in Follicular Fluid: Key Molecular Signatures

Studies investigating FF biomarkers have primarily analyzed the profile of proand anti-inflammatory cytokines within the FF environment. Among these cytokines, Molka *et al.* (2022) observed a significant increase in pro-inflammatory cytokine IL-6 levels among patients aged ≥ 35 or with a history of embryo development failure or recurrent implantation failure (RIF). It is notable that IL-6 not only regulates the proliferation and apoptosis of GC but also plays a role in follicular dynamics. Specifically, elevated IL-6 levels have been linked to increased preantral follicle survival (Yang *et al.*, 2020), suggesting that higher IL-6 concentrations could enhance GC viability, thereby potentially influencing oocyte quality. Bouet *et al.* (2020) further identified variations in cytokine levels, such as IL-2, IL-8, and IL-10, in the follicular fluid of older women and poor ovarian responders, highlighting the complexity of cytokine interactions in ovarian ageing. Moreover, their study pointed out a significant decrease in PDGF-BB concentration in diminished ovarian reserve patients, which may be linked to increased oxidative stress and altered folliculogenesis.

Concurrently, tumor necrosis factor-alpha (TNF-α) has previously been shown to enhance folliculogenesis and ovulation by promoting vascularization and suppressing GC proliferation and spontaneous apoptosis (Wyse *et al.*, 2021). In this way, inflammatory profiles seem to impact oocyte development, with IL-10 mediating communication between oocytes and GC, potentially affecting the intrafollicular environment during maturation (Jatesada *et al.*, 2013; Kollmann *et al.*, 2017; Lee, 2021). Consistently, Wyse *et al.* (2021) identified TNF-α as a reliable predictor of oocyte maturation rate across the examined cohort. However, when data was stratified by BMI, IL-10 showed a stronger predictive capacity for oocyte maturation in normal-weight patients. Such data suggest that their predictive efficacy may vary depending on patient characteristics.

Estradiol (E2) also plays a critical role in oocyte development, particularly during nuclear and cytoplasmic maturation Uppangala *et al.* (2020). Synthesized by GC, E2 influences the cellular redox state, exhibiting both pro and antioxidant properties. In this context, insufficient serum E2 levels may compromise the protection against oxidative damage to the COC in poor responders (Uppangala *et al.*, 2020). Furthermore, an intra-follicular environment lacking both E2 and melatonin negatively impacts embryo quality and fragmentation rate Latif Khan *et al.* (2020). However, Uppangala *et al.* (2020) did not assess E2 levels in the FF, and they do not solely attribute oxidative stress to inadequate E2. Carpintero *et al.* (2014) emphasized the importance of a balanced hormonal environment in follicular fluid, noting that higher estradiol levels are associated with better embryo quality and higher pregnancy rates. This supports the notion that E2 plays a crucial role in creating an optimal environment for oocyte maturation and successful fertilization.

Similarly, Bastu *et al.* (2015) found that high levels of cathepsin B in follicular fluid correlated with higher pregnancy rates, suggesting its involvement in oocyte maturation and embryo development. Meijide *et al.* (2017) also highlighted the importance of antioxidant enzymes, such as PONs, in follicular development, noting increased PON activities in larger follicles. However, further studies are necessary to fully understand the role of PONs in human reproduction.

GH has been the subject of numerous studies (Ipsa *et al.*, 2019; Scheffler *et al.*, 2021; Stoecklein *et al.*, 2021; Molka *et al.*, 2022; He *et al.*, 2023) due to its multifaceted role in enhancing oocyte quality. Specifically, GH has been shown to increase the FSH and LH receptors of follicles and improve oocyte mitochondrial function (Molka *et al.*, 2022; Yu *et al.*, 2022). Yu *et al.* (2022) established a significant correlation between FF LH, FSH, cortisone levels, and oocyte development. Additionally, GH positively regulates the expression of insulin growth factor I (IGF1), thereby promoting follicle growth in patients with suboptimal responses to treatment (Scheffler *et al.*, 2021). IGF1, in turn, plays a crucial role in ovarian function, follicular development, estrogen production, and oocyte maturation (Scheffler *et al.*, 2021). Notably, Molka *et al.* (2022) established a correlation between IL-6 and IGF1 levels, indicating potential crosstalk between inflammatory and growth factor signaling pathways in the follicular environment. Interestingly, IGF1 was also found to be linked to GH levels (Molka *et al.*, 2022). These interconnections underscore the relationship between GH, IGF1, and the ovary, offering alternatives to improve strategies for patients with poor responses to ovarian stimulation.

Melatonin, a potential biomarker of oocyte maturation (Latif Khan *et al.*, 2020; Zhang *et al.*, 2021b), has been shown to maintain mitochondrial membrane potential, regulate Ca2+ levels, suppress oxidative stress, and preserve oolemma permeability (Liu *et al.*, 2019). In this scope, Latif Khan *et al.* (2020) found a negative correlation between melatonin concentration and cfDNA levels. Similarly, Debbarh *et al.* (2023) established a correlation between higher levels of cfDNA and reduced oocyte maturity and embryo quality.

Vitamin D also plays a role in oocyte maturation, influencing follicular physiology through mechanisms such as promoting cell proliferation, modulating inflammatory responses, and increasing E2 and progesterone levels (Ekapatria *et al.*, 2022). However, the relationship between follicular vitamin D concentrations and oocyte quality remains inconsistent across studies. Ekapatria *et al.* (2022) reported a significant positive correlation, with oocyte quality being higher in patients with vitamin D levels ≥13.7ng/mL (n=39) compared to those with lower levels. In contrast, Ciepiela *et al.* (2018) observed a negative correlation, categorizing patients into <20ng/mL (n=114) and ≥20ng/mL (n=84), and reporting lower follicular vitamin D concentrations in successfully fertilized oocytes compared to those that failed fertilization. These discrepancies may stem from differences in threshold values used to classify vitamin D status, as well as variations in patient selection criteria across studies.

Previous studies have demonstrated a correlation between serum and follicular vitamin D levels, consistently reporting that follicular concentrations tend to be higher than in serum (Ozkan *et al.*, 2010; Aleyasin *et al.*, 2011; Firouzabadi *et al.*, 2014). Importantly, these studies did not exclude patients with PCOS as a cause of infertility, differing from the selection criteria applied in this review. However, Aleyasin *et al.* (2011) reported no significant variation in serum and follicular vitamin D levels across causes of infertility. More recently, Baldini *et al.* (2024) investigated the impact of vitamin D supplementation combined with myo-inositol, folic acid, and melatonin, demonstrating that increasing serum and follicular vitamin D levels prior to ART was associated with improved embryo quality. These findings suggest that vitamin D supplementation may represent a promising therapeutic approach in ART; however, further investigations are warranted to clarify its role in follicular physiology and oocyte and embryo quality (Ciepiela *et al.*, 2018; Ciepiela, 2019).

### Additional Parameters of Oocyte Quality Assessment

#### Patient-Related Factors

Several physiological factors, including age, ovarian reserve, and BMI, influence both oocyte and embryo quality. Age-related declines in mitochondrial function and alterations in GC gene expression have been well-documented (Shen *et al.*, 2020; Cecchino *et al.*, 2021), while antral follicle count (AFC) and anti-Müllerian hormone (AMH) remain established indicators of oocyte competence (Broekmans *et al.*, 2006; Jayaprakasan *et al.*, 2010; La Marca *et al.*, 2010; Scantamburlo *et al.*, 2021). BMI, however, has a more complex association with reproductive outcomes. Some studies suggest that it modulates inflammatory cytokine activity (Wyse *et al.*, 2021) and affects oocyte metabolism and maturation, with gene expression variations in cumulus cells influencing proliferation and glycolytic activity (Shen *et al.*, 2020). Others, however, have found no significant impact of BMI on oocyte quality or ART success (Banker *et al.*, 2017; Sheng *et al.*, 2017; Maged *et al.*, 2019), emphasizing the need for further investigation into these mechanisms (Snider & Wood, 2019).

Beyond female factors, sperm quality also plays a critical role in fertilization and embryo development. Male infertility has been associated with oxidative stress, DNA fragmentation, and epigenetic alterations, all of which may compromise embryonic viability (Takeshima *et al.*, 2020). However, comparing outcomes across studies remains challenging due to variability in patient selection criteria, as some apply strict inclusion criteria by setting different limits for BMI and age, as well as varying inclusion and exclusion criteria for male infertility, while others adopt a more inclusive approach without these restrictions, potentially increasing heterogeneity in reported fertilization rates and embryo quality. These inconsistencies highlight the need for standardized selection criteria in future research to improve comparability and refine biomarker analysis.

#### Advancements in Non-Invasive Technologies

Numerous studies reaffirm that morphological assessment plays an essential role in optimizing ART outcomes (Figueira *et al.*, 2010; Lasiene *et al.*, 2011; Sciorio *et al.*, 2024). Oocyte morphology has been evaluated based on attributes such as size, cytoplasm, zona pellucida, perivitelline space, and polar body (Ekapatria *et al.*, 2022). Additionally, Bartolacci *et al.* (2022) highlighted the relevance of COC morphology, vacuoles, smooth endoplasmic reticulum aggregates, and granularity in predicting developmental competence. However, despite its clinical relevance, morphological assessment may be influenced by subjective interpretation Manna *et al.* (2013). To address this limitation, data automation tools have been introduced as complementary methods to refine oocyte evaluation (Fischer *et al.*, 2021; Chéles *et al.*, 2022), enhancing objectivity and reproducibility in clinical practice.

Integrating molecular and imaging-based techniques, non-invasive approaches have emerged as promising tools for evaluating oocyte competence (Sciorio *et al.*, 2022). Optical-based methods, such as polarized light microscopy and refractive index measurements, have been applied to assess oocyte structures, while time-lapse embryo culture technology enables continuous imaging of embryo development (Marquet *et al.*, 2005; Zeng *et al.*, 2024). Additionally, artificial intelligence-driven models integrate imaging data with clinical factors to enhance predictive accuracy (Zeng *et al.*, 2024).

At a molecular level, EV-miRNAs have been identified as key players in ovarian follicle communication, influencing granulosa cell function, follicular development, and oocyte maturation (Andronico *et al.*, 2019; Martinez *et al.*, 2019; Qasemi & Amidi, 2020; Shen *et al.*, 2020; Zhang *et al.*, 2021a). Their profiles are modulated by stimulation protocols, suggesting potential implications for IVF success (Machtinger *et al.*, 2023). These molecular biomarkers candidates, alongside imaging-based approaches, contribute to a more comprehensive and non-invasive evaluation of oocyte quality. Although these techniques offer valuable insights into oocyte and embryo viability, further validation is required to establish their clinical applicability and standardization.

### Strengths and Limitations

Our study has certain limitations related to the variability among the selected studies. Differences in inclusion and exclusion criteria led to heterogeneity in patient demographics, infertility management protocols, and the consideration of male factor infertility, which may affect the generalizability of the findings. Beyond demographic factors, variability in ovarian stimulation protocols, timing of human chorionic gonadotropin administration, and criteria related to hormonal disorders or mitochondrial function further contributed to methodological heterogeneity. These differences reflect the diverse approaches used to assess biomarkers of oocyte and embryo quality, highlighting the complexity of consolidating findings across studies. Additionally, small sample sizes and study design biases, such as retrospective analyses and methodological inconsistencies, may have influenced the reported associations between biomarkers and reproductive outcomes.

However, one of the key strengths of this review is the minimized clinical impact of PCOS and severe endometriosis on oocyte evaluation. By excluding studies involving patients with these diagnoses, we ensured that the results were not biased by these clinical conditions. Furthermore, the predominant inclusion of prospective studies reduces reliance on tertiary data, enhancing the reliability of the findings. Despite the inherent variability across studies, this review provides valuable insights for reproductive medicine professionals by refining biomarker-based assessments. Future research should focus on larger, well-controlled prospective studies to validate these findings and establish standardized clinical applications, ultimately contributing to the improvement of ART outcomes.

## CONCLUSION AND FUTURE DIRECTIONS

Most articles identified significant associations between predictors in FF and GC with oocyte quality. In FF, levels of specific cytokines, melatonin, GH, IGF1, and TNF-α were considered relevant biomarkers (Latif Khan *et al.*, 2020; Wyse *et al.*, 2021; Molka *et al.*, 2022; Yu *et al.*, 2022). In GC, bioenergetic properties, proand anti-inflammatory mediators, and specific transcriptomic signatures have been identified as influential predictors in the assessment of oocyte quality (Martinez *et al.*, 2019; Scarica *et al.*, 2019; Karabulut *et al.*, 2020; Shen *et al.*, 2020; Uppangala *et al.*, 2020; Wyse *et al.*, 2020; Daei-Farshbaf *et al.*, 2021; Martins *et al.*, 2022). Additionally, morphological evaluations (McCulloh *et al.*, 2020; Bartolacci *et al.*, 2022; Ekapatria *et al.*, 2022; Tepla *et al.*, 2022) and EV-miRNA expression profiles (Martinez *et al.*, 2019; Zhang *et al.*, 2021a; Machtinger *et al.*, 2023) have emerged as promising tools for assessing oocyte maturity, quality, and overall reproductive potential.

The identification of multiple factors as potential predictors of oocyte quality highlights the complexity of this process. Our review underscores FF oxidative stress evaluation and GC transcriptomic signatures as promising biomarkers for oocyte assessment, with potential relevance in clinical settings. Advances in non-invasive technologies, including genomics, molecular profiling and artificial intelligence-assisted evaluation, may further enhance biomarker-based assessments, refining oocyte selection criteria and optimizing ART success rates. To ensure their effective clinical implementation, additional research is needed to validate these approaches and establish standardized protocols.
